# Towards Improved Use of Vaccination in the Control of Infectious Bronchitis and Newcastle Disease in Poultry: Understanding the Immunological Mechanisms

**DOI:** 10.3390/vaccines9010020

**Published:** 2021-01-04

**Authors:** Anthony C. Ike, Chukwuebuka M. Ononugbo, Okechukwu J. Obi, Chisom J. Onu, Chinasa V. Olovo, Sophia O. Muo, Okoro S. Chukwu, Eleazar E. Reward, Odinakachukwu P. Omeke

**Affiliations:** 1 Department of Microbiology, Faculty of Biological Sciences, University of Nigeria, Nsukka, Enugu 410001, Nigeria; obi.okey@yahoo.com (O.J.O.); chisomjoshua982@yahoo.com (C.J.O.); chinasa.olovo@unn.edu.ng (C.V.O.); muosophia0@gmail.com (S.O.M.); cokorosamuel@gmail.com (O.S.C.); omekeprisca123@gmail.com (O.P.O.); 2 Department of Biotechnology, Graduate School of Engineering, Osaka University, Suita City, Osaka 565-0871, Japan; ononugbocm@gmail.com; 3 Department of Biological Sciences, College of Liberal Arts & Sciences, Wayne State University, Detroit, MI 48202, USA; reward.eleazar@wayne.edu; 4 School of Medicine, Clinical Medicine Major, Jiangsu University, Zhenjiang 212013, China; 5 Department of Biology/Microbiology/Biotechnology, Alex Ekwueme Federal University Ndufu Alike, Ebonyi 1010, Nigeria

**Keywords:** infectious bronchitis, Newcastle disease, poultry, vaccine, immunity

## Abstract

Infectious bronchitis (IB) and Newcastle disease (ND) are two important diseases of poultry and have remained a threat to the development of the poultry industry in many parts of the world. The immunology of avian has been well studied and numerous vaccines have been developed against the two viruses. Most of these vaccines are either inactivated vaccines or live attenuated vaccines. Inactivated vaccines induce weak cellular immune responses and require priming with live or other types of vaccines. Advanced technology has been used to produce several types of vaccines that can initiate prime immune responses. However, as a result of rapid genetic variations, the control of these two viral infections through vaccination has remained a challenge. Using various strategies such as combination of live attenuated and inactivated vaccines, development of IB/ND vaccines, use of DNA vaccines and transgenic plant vaccines, the problem is being surmounted. It is hoped that with increasing understanding of the immunological mechanisms in birds that are used in fighting these viruses, a more successful control of the diseases will be achieved. This will go a long way in contributing to global food security and the economic development of many developing countries, given the role of poultry in the attainment of these goals.

## 1. Infectious Bronchitis (IB) and Newcastle Diseases (ND) in Poultry

### 1.1. Introduction

Infectious bronchitis (IB) and Newcastle disease (ND) are among the most important viral diseases of poultry with substantial global economic impact [1,2]. Infectious bronchitis is caused by the IB virus (IBV), a member of the Gammacoronavirus genus, family Coronaviridae, and subfamily Orthocoronavirinae. IBV is commonly referred to as avian coronavirus and it belongs in the same family and subfamily as the severe acute respiratory syndrome coronavirus 2 (SARS-CoV-2), which is currently ravaging the world, although the latter is in a different genus—Betacoronavirus. Newcastle disease (ND) is caused by ND virus (NDV), which belongs to the genus Avulavirus in the family Paramyxoviridae. Both viruses have genomes made up of single stranded RNA (ssRNA). However, while the RNA of IBV is positively stranded, that of NDV is negatively stranded. Due to the single stranded nature of their genome, these two viruses are able to evolve rapidly, leading to high genetic variability in circulating virus strains [3,4]. This is even more pronounced in the case of IB, where recombination contributes to genetic variation [5]. IB and ND affect poultry birds of all ages and breeds, but the degree of disease varies based on the age of the birds, with IB being more severe in young chicks [1,6] and the severity of ND more pronounced in chickens of all ages. In addition, the main immunoglobulin in avian is IgY, an immunoglobin similar in function and concentration to IgG in mammals and previously called by the same name. However, this has been found to be incorrect as there are clear differences in molecular structure between the two immunoglobulins [7]. In this review we have used IgY in place of IgG when discussing avian immunology. The main types of vaccines used against these viruses are live attenuated and inactivated vaccines. Live attenuated vaccines pose the risk of spreading live viruses, while inactivated vaccines are generally of low immunogenicity. Additionally, control of IBV through vaccination is hindered by the continuous mutation in the spike (S) protein, which results in antigenic variation and circulation of different serotypes, which do not provide cross-protection [8]. Although all NDV strains are of one serotype, substantial genetic diversity exists [9], and different isolates may not provide complete cross-protection against each other [10,11]. As a result of these variations, there is a need for continuous vaccine development against these viruses. Other major challenges encountered in vaccination against ND are those of handling and storage, especially in the tropics [12]. In this review, we examine the development of vaccines against these important poultry pathogens and how the knowledge of the immunological mechanisms in birds is contributing to this.

### 1.2. Public Health Importance of IB and ND in Poultry

Infectious bronchitis (IB) and Newcastle disease (ND) affect poultry birds of all ages and breeds. The extent and severity of IB is pronounced in young chicks [1], when compared to older chicken, while ND though seen in chickens of all ages, has a more protracted course in older birds [13]. Crinion and Hofstad [6] reported that the severity of IB lesions is more pronounced in day-old chicks and decreased with increase in age of the chickens.

Humans are susceptible to Newcastle disease virus (NDV). This may cause mild conjunctivitis, reddening of the eye, excessive lachrymation, edema of the eyelids, sub-conjunctival hemorrhage, and laryngitis when exposed to large doses of the virus [14,15]. NDV-associated conjunctivitis usually resolves rapidly, but the virus could be shed in the ocular discharge for about a week. In some cases, mild, self-limiting flu-like disease could result. Other clinical symptoms such as chills, headaches, and fever, with or without conjunctivitis, may occur. Both the strains used in vaccines and virulent strains associated with poultry may infect and cause clinical signs in humans. Laboratory workers and vaccinators are mostly affected, but the use of personal protective equipment and bio safety cabinet has significantly minimized the exposure of laboratory workers. Infection is rarely seen in farm workers [16]. There is no evidence to support human-to-human transmission, but the potential for human to bird transmission exists [17]. Newcastle disease virus does not pose a risk to food safety.

Infectious bronchitis virus (IBV) poses no risk to human health as there is no evidence to suggest that humans act as reservoir for active replication of IBV. Also, there is no evidence of transmission from human to human, or human to animal. Though neutralizing antibodies have been documented in people working with commercial chicken flocks [18], the significance of the neutralizing antibodies remain unknown. Humans can only transmit IBV to poultry birds by mechanical means, by carrying virus on clothing from an infected bird or flock to uninfected ones. A reported isolation of an avian IBV-like virus from humans was later shown to be a human Coronavirus isolate [18].

### 1.3. Economic Importance of IB and ND in Poultry

Infectious bronchitis virus (IBV) and Newcastle disease virus (NDV) cause devastating economic loss in poultry industries worldwide. Poultry are among the largest livestock group and yields more than 30% of all animal protein [19]. Poultry meat is a significant protein source in peoples’ diet because of its affordability and high protein yield [20].

Infectious bronchitis-associated morbidity can reach up to 100% in a flock. Mortality rates ranges from 25 to 30% in young chicks and could reach up to 80% as a result of host-associated (age, immune status), virus-associated (strain, pathogenicity, virulence, and tissue tropism), or environmental-related (cold and heat stresses, dust, and presence of ammonia) factors [1].

Economic losses arise due to the direct impact of IBV and NDV on poultry birds. The management practices adopted in poultries and the infecting viral strain determine the nature of the economic impact. Infectious bronchitis (IB) is a debilitating disease that results in poor utilization of feed by young birds resulting in poor weight gains, air sacculitis, leading to condemnation and colossal waste during processing. An estimated 8% of poultry birds can be condemned at the processing plant due to an outbreak of IB, compared to 1% in IBV controlled poultry [18]. Delayed maturity, production inefficiencies during infection and post-infection result in over 70% reduction in egg-laying potential of the poultry birds [21,22]. In highly susceptible birds, gains in egg production could be revived, but the peak rate may be permanently depressed. Fertility rates could be greatly impacted during an outbreak/post-outbreak. In addition to losses from poor weight gain and depreciated carcasses, losses in the order of 10% to 25% mortality may be recorded in cases of infectious bronchitis nephritis [18].

A reduction in income by approximately 3% in an IBV-infected commercial flock with the best possible management practices have been estimated by McMartin [23] in comparison with a hypothetical flock free from infectious bronchitis virus. Assayad et al. [24] documented economic losses of US$251.4 per 1000 birds and US$8.46/1000 birds in Poultry breeders and broilers respectively. Whereas Colvero et al. [25] reported economic losses of US$3567.4/1000 birds and US$4210.8/10,000 birds for 25–26- and 42 weeks--old breeders respectively, and US$266.3/1000 birds in 48 days-old broilers. IBV is rated among the biggest single cause of infectious disease-related economic loss [26], and this is usually more pronounced in developing regions where effective poultry management and proper vaccinations are not implemented.

Newcastle disease is a silent killer that kills millions of village (backyard) chickens and takes away the source of livelihood of poor rural women. Newcastle disease can drastically limit the amount of dietary protein as well as the micro-economy (loss of market values for the birds and eggs) in developing countries where the majority of chickens are reared under subsistence farming conditions. ND outbreaks have also occurred in commercial poultry farms across the globe resulting in massive economic damages. ND has been described as the single greatest constraint on the production of village poultry [27]. In areas where ND is endemic, it could discourage farmers from investing time and money in commercial poultry husbandry. It has been claimed that ND constitutes a colossal drain on the world economy compared to other animal viral diseases [28], most of which result from the control measures and trade losses. For instance, during the last major outbreak of ND in the United States, in California in 2002–2003, about 2500 premises consisting of 4 million birds were slaughtered at a cost of approximately US$162 million [19]. Infection of village chickens with NDV in Bangladesh in early/mid-2014 led to the deaths of about 100,000 birds and, on average, the country is estimated to suffer economic losses in the tune of US$288.49 million per annum [28]. ND has been associated with a mortality rate of over 55% in Chad, with a loss estimated at more than US$64 million [29]. In a study carried out in Abuja, Nigeria, the outbreaks of ND resulted in the highest cumulative loss of about US$50,000 compared to other viral diseases such as IB and avian influenza [30]. These economic losses usually arise from depreciation in birds’ value, mortality, and job losses/management cost [31]. The vaccination costs are significant and could be a setback in some developing countries. Also, the massive use of vaccines (live and inactivated) explains the huge economic importance attached to NDV and IBV.

Unless effective measures are instituted, decline in egg production as a result of the viral diseases does not return to normal laying, thus contributing to high economic loss [32]. It should be highlighted that there is a paucity of information on economic losses, especially in developing countries (endemic regions) due to the subsistence nature of the farming system and under-reporting of outbreaks.

## 2. Immunity and Immune Response against IB and ND in Poultry

### 2.1. Immune System in Birds Relative to Viral Diseases

Just like all vertebrates, Aves (birds) have two complementary immune systems, the innate and adaptive immune systems. Together with proteins, cells, and complementing molecules, they function to block pathways that lead to viral infections [33]. Due to the persistent nature of the viruses that cause IB and ND, the chicken immune system has been extensively studied to understand their importance in viral immunity and identify markers for effective vaccine development. In as much as there are many similarities between the chicken immune system and those of mammals, there are significant outstanding differences as well [34,35,36]. The avian immune system presents heterophils as its equivalent to mammalian neutrophils. They lack functional eosinophils and lymph nodes. Although the lymphatic system is also categorized into primary and secondary organs [37], chickens possess two central (primary) lymphoid organs; the thymus and the bursa. Chicken susceptibility to viral infection is influenced by their major histocompatibility complex (MHC I & II) genotype. MHC determines the quality of humoral and cell-mediated responses, as the MHC molecule is responsible for binding the antigen epitope and to present it to T lymphocytes [38,39].

The bursa of fabricius is responsible for the functioning of humoral, non-cellular or antibody-mediated immune response in chickens. Prebursal stem cells migrate into the bursa and differentiate into bursa stem cells, these bursa stem cells develop into post-bursal B cells. The Antigen-specificity of the developed B cells involved in humoral immunity is developed during the differentiation process in the bursa [40]. Antigen-specific property of B cells enables them to recognize antigens based on specific antibody molecules. There are only three antibodies in chicken immunology: IgY, IgA, and IgM [7,41]. In avian IB and ND, the associated local antibodies present on the mucosa-associated lymphoid organs (MALT) of the respiratory tract are IgA and IgY [42,43]. Generally, MALTs in chickens associated with IBV and NDV include nasal-associated lymphoid tissues (NALT) and bronchus-associated lymphoid tissue (BALT) of the lungs [44,45]. The NALT play their role efficiently due to the presence of the areas of B cells with caps composed of CD4+ T cells. The B cells are distributed all around the epithelium of the nasal cavity [46,47].

The thymus gland is the site for T cell lymphocyte production and development. The T cell is the effector cell in cell-mediated immune response. There are two types of T cell; γδ and αβ, which have their specific T cell receptor (TCR). T cells that primarily play a regulatory role in adaptive immunity, whether cell-mediated or humoral, are referred to as T helper (Th) cells and typically express CD4 molecules on their surface [48]. There are three types of helper T cells associated with the chicken immune system: Th1, Th2, and Th17 [49].

### 2.2. Host Immune Responses to IB and ND

#### 2.2.1. Host Immune Responses to IBV

**Figure 1:**
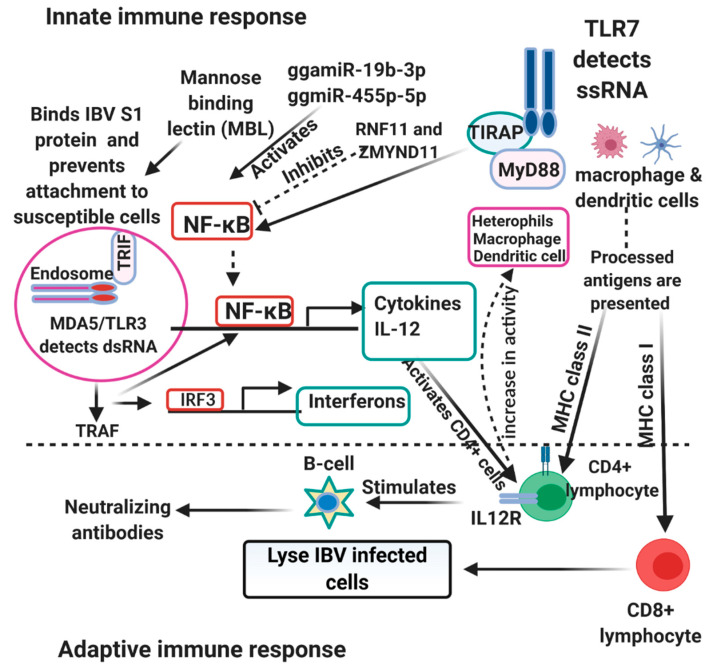
Host immune response to Infectious bronchitis virus (IBV) and Newcastle disease virus (NDV). The Toll-like receptors 4, 15, and 16 on macrophages, dendritic cell and heterophils sense and activate the NF-κB which leads to production of cytokines. Peptides from the virus is also processed and presented to the adaptive immune cells (CD4+/CD8+) by the antigen-presenting cells (APCs) in the company of the major histocompatibility complex II/I (MHCII/MHCI). The adaptive immune cells stimulate the production of neutralizing antibodies and stimulate interferon production which also increases the phagocytic ability of macrophages, dendritic cells and heterophils. NDV inhibits NF-κB by RNF11 and ZMYND11, while the miRNA ggmiR-19b-3p activates NF-κB.

The innate immune system is the first line of defense against viral infections and plays a very important role in non-specific immunity and activation of specific immunity against infections. The innate immune response is activated upon detection of invading pathogens and determines the outcome of infections. Activation of the innate immune response relies upon the recognition of pathogen-associated molecular patterns (PAMPs) by specific pattern-recognition receptors (PRRs). Similar to findings in humans, Toll-like receptors (TLRs) and RIG-1-like receptors (RLRs) are the two main PRRs involved in the recognition of viral components [50]. There are several TLRs upregulated in response to viral infections (TLR4, TLR5, TLR15, TLR16) [51]. However, TLR3 recognizes viral dsRNA and TLR7 recognize viral ssRNA. On the recognition of their respective targets, TLR3 recruits the adaptor TIR domain-containing adaptor inducing interferon (IFN)-β (TRIF) while TLR7 recruits myeloid differentiation primary response 88 (MyD88). The recruited TRIF interacts with tumor necrosis factor (TNF) receptor-associated factor (TRAF) 3 and TRAF6. TRAF6 either directly phosphorylate IFN regulatory factor 7 (IRF7) or activate the transforming growth factor B-activated kinase 1 (TAK1). The activated TAK1 binds to the inhibitor of kappa B kinase (IKK) complex and activate the nuclear factor of kappa B (NF-κB). The activated NF-κB moves from the cytoplasm to the nucleus and induce the expression of inflammatory cytokines and chemokines [52]. TRAF-3, on the other hand, recruits the IKK-related kinases TANK-binding kinase 1 (TBK1) and IKKε which phosphorylates IRF7. The phosphorylated IRF7, a transcriptional activator, moves into the nucleus and binds to the interferon-stimulated response element (ISRE) on interferon (IFN) promoters and transcriptionally activate type I IFN (IFN-α and IFN-β) and IFN-inducible gene expression [52,53]. RLRs (RIG-1 and melanoma differentiation-associated protein 5 (MDA5 or IFIH1)) senses viral dsRNA in the cytoplasm to induce antiviral response and are greatly induced by type 1 IFN [54]. Both RIG-1 and MDA5 recognizes different viral RNA intermediates. RIG-1 recognizes short dsRNA and uncapped 5′ triphosphate ssRNA, while MDA5 recognizes long dsRNA. As RIG-1 is absent in chickens, MDA5 is the major RLR in detecting viral nucleic acid in the cytoplasm of chickens and it is under the regulation of laboratory of genetics and physiology 2 (LGP2) [54,55]. When MDA5 is activated, it interacts with adaptor mitochondrial antiviral signaling protein (MAVS) (also known as IPS-1, CARDIF, or VISA) and activates it. The activated MAVS causes recruitment of TRAF6 and TRAF3 leading to the assembly of IKKε and TBK1 complex, causing the activation of NF-κB and IRF7 and subsequent production of pro-inflammatory cytokines and type I IFN [52,56]. More so, IRF1, IRF8, IRF10 and ISGs (OSAL, MX1, IFIT5, ISG12-2, RSAD2, IFI35, protein kinase R (PKR) and IFI27L2) were upregulated in IBV-infected birds and activated Janus kinase/signal transducer and activator of transcription (JAK-STAT) pathway induces transcription of ISGs [51]. Among the activated Type I IFNs, IFN-β plays a dominant role in resisting IBV infection. Furthermore, despite IFN-γ having weak antiviral activities, it enhances the immune response by activating macrophages, T cells and plays a role in tracheal lesions, while IFN-λ enhance host resistance to IBV [57]. Interleukin 1β, one of the pro-inflammatory cytokines binds to cell surface receptors on infected cells and initiates recruitment of several immune and inflammatory cells including macrophages, dendritic cells, and other antigen-presenting cells (APCs) to the site of infection. Activated macrophages initiate inducible nitric oxide synthases (iNOS), which catalyze the production of nitric oxide (NO), inducing antiviral responses [58]. Macrophages, which are important for phagocytosis, also produces cytokines (Interleukin 1β), and as APCs initiate antigen-specific adaptive immune response. This is illustrated in Figure 1. Infected macrophages are destroyed through apoptosis [58]. Avian β-defensins (AvBDs), cathelicidins (CATHs) and Prostagladin (PG) E2 (PGE2) are also induced by IBV antigens. Moreover, PEGS further induce production of IFNs and AvBDs [59]. It is thought that microbial ligand-mediated induction of TLR signaling also induces the expression of defensins and AvBDs play an important role in the defense against IBV replication. AvBDs activate immature dendritic cells and antigen-presenting cells (APCs) through interaction with TLRs [60]. Generally, IBV infection induces increased expression of IL6, IL18, IL10RA, IL17RA, CCL4, CCL20, CCL17, and CCL19. The upregulation of these chemokines might attract the immune cells to the site of infection and leads to increase in synthesis of IL-6 [51,61]. IL-6 activates lymphocytes, leukocytes, and eosinophils infiltration to the site of infection and upregulation of SOCS-1 and SOCS-3 expression in IBV-infected cells serve as the feedback system of IL6 signal transduction [61]. MHC I was also upregulated upon IBV infection and plays significant roles in viral clearance [51]. The upregulation of CD38, CD40, CD80, CD86 markers on T cells and APCs on interaction becomes fully activated and releases co-stimulatory molecules. Activated T lymphocytes differentiate into cytotoxic T cell, T helper cells, and subsequently activate the B cells for anti-IBV immunoglobulin production and subsequent viral clearance [53,62]. Prior to the onset of humoral response, IBV also induces robust specific CTL activity during acute infection. The kinetics of the viral load correlates with the level of this IBV-specific CTL activity and is responsible for the initial decline in viral load at the early stage of acute infection [63]. Seo and Collisson also showed that the specific lysis of the IBV-infected cells were mediated by CD8^+^ CD4^−^ T cells rather than CD4^+^ CD8^−^ T cells [63]. Mannan-binding lectin (MBL), a c-type collection and a pattern-recognition molecule in innate immunity binds selectively to carbohydrates expressed on the surface of all kinds of pathogens, and chicken MBL binds to IBV spike protein through its carbohydrate recognition domain (CRD) aggregating the virus particle [64]. It also acts as an opsonin and mediates lysis and phagocytosis of IBV through the lectin pathway of the complement system [64].

#### 2.2.2. Host Immune Responses to NDV

Toll-like receptors upon interaction with PAMPs get activated and lead to subsequent activation of IRF1 and IRF3 and the production of type 1 IFNs. In chickens, several TLRs have been identified. However, among these, TLR3 recognizes dsRNA as a replication intermediate, while TLR7 recognizes ssRNA [65,66]. TLR3 upon recognition of its target, triggers downstream cascade signals through the Toll-IL-1 receptor (TIR) domain-containing adaptor-inducing IFN-β (TRIF) and this interaction activates TANK-binding kinase 1 (TBK1) and inducible IκB kinase (IKK-i). The activated kinases (TBK1 and IKK-i) directly phosphorylate IRF3 and IRF7. IRF3 and IRF7 upon phosphorylation, translocate to the nucleus and activate the transcription of type 1 IFNs and IFN-inducible genes. TLR7 on the other hand, activate IRF-7 through MYD88, IL-IR-associated kinase 1 (IRAK1) and IKK-α, but not through TBK1 or IKK-I [67]. MDA5, an RLR recognizes both long and short viral dsRNA in the cytoplasm and recruit MAVS (also known as IPS-1, VISA, or CARDIF) to activate downstream cascade signals for production of type 1 IFNs and pro-inflammatory cytokines through IRFs and nuclear factor kappa B (NF-κB) for its antiviral functions [68]. More so, IFN-α and IFN-β activate the JAK/STAT cascade signals in both paracrine and autocrine manners, which further stimulate ISGs to arrest replication of the viruses [69]. Additionally, LPG2 is believed to play a role in potent antiviral response in chickens via communication with MDA5 [70]. However, the exact mechanism is not clear. The overexpression of IPS-1 induces the production of pro-apoptotic genes (TRAIL) and downregulates the production of anti-apoptotic genes (BCL2, BIRC3 and PRKCE) through IRF3 and IRF7 [68]. AvBD2 is also produced downstream of the TLRs and it is under the regulation of p38 MAPK [71]. p38 MAPK is one of the three subfamilies (c-Jun N-terminal kinase (JNK) and extracellular signal-regulated kinase ½ (ERK ½)) of the MAPK family of serine/threonine kinases and it is activated down steam of the TLRs [71]. IFNs and other cytokines activate the natural killer cells, dendritic cells (DCs), macrophages to limit the replication of viruses via the adaptive response. Several other genes including other ISGs (Mx, OAS, PKR), IRF1, STAT1, IFN- γ, CCL5, IL-8, IL-6, IL-12 α, IL-6, and IL-1β are also upregulated in response to NDV infection as reported by some studies [65,70,72,73,74] and could contribute in innate signaling pathways or directly stimulate immune cells for antiviral function. IL-6, a cytokine produced from activated endothelial cells, fibroblasts, and macrophages promotes the differentiation and activation of B and T lymphocytes. It further recruits inflammatory cells to the site of infection and stimulates the synthesis of prostaglandin E2 from endothelial cells, which binds to the receptor EP3 in neural cells to generate fever. IFN-γ stimulates the responses of Th-1 cells, dendritic cells and macrophages leading to the production of IFN-β, upregulation of the expression of MHC I and II molecules, antigen presentation, and processing [70,75]. Therefore IFN-γ could be very useful in arresting viral replication through immune cells stimulation. Activation of protein kinase R (PKR) by dsRNA generated by NDV infection replication intermediate inhibits NDV replication via the PKR/eukaryotic translation initiation factor 2 (eIF2) cascade signal. eIF2α on phosphorylation terminates translation at the global level resulting in the activation of apoptosis pathways in NDV-infected cells to limit viral spread [76,77]. Viperin an IFN-inducible protein reduces NDV replication through interacting with the matrix protein of the virus [78]. NDV infection induces upregulation of chicken-galectin 1B, which binds to hemagglutinin-neuraminidase (HN) on the virus, preventing the activity of the hemagglutinin [79]. NDV inhibits NF-κB signaling using ring finger protein 11 (RNF11) and zinc-finger protein, MYND-type containing 11 (ZMYND11), siRNA negative regulators of NF-κB suppressing production of inflammatory cytokines in DF-1 cells, two negative regulators of NF-κB signaling, in DF-1 cells. Infection of chicken dendritic derived cell with virulent NDV elicited MD5, TLR3, TLR7, interferons, IL1β, IL-6, IL-18, 1L-10, and IL-12 production [80] and the relative levels of expressions depend on the strain pathogenicity. gga-miR-19b-3p stimulates NF-κB signaling by binding the mRNA of both RNF11 and ZMYND11 [81]. Gga-miR-455-5p increases IFN-β IRF3, Mx1, and OASL and the knockdown suppresses their expression (Figure 1).

### 2.3. Vaccine-Mediated Immunity against IB and ND

#### 2.3.1. Vaccine-Mediated Immunity against IBV

Live attenuated vaccines against IBV infection induce both humoral and cellular immunity in immunized hosts as demonstrated by several studies. Vaccination of chickens with live H120 alone or in combination with live CR88 induced high titers of IgY and IgA anti-IBV antibodies, CD4+ T cells, CD8+ T cells, and granzyme homolog A [82,83]. LDT3-A and 4/91 commercial live vaccines induced production of antibodies and CD4+ and CD8+ T cells in vaccinated birds [84]. Ocular vaccination with live attenuated IBV Ark-Delmarva industry vaccine induces IgA anti-IBV antibody as a primary response to infection while memory response to infection is dominated by IgY [85]. Vaccination of day-old broiler chicks with Massachusetts (Mass), 793B, D274, or Arkansas (Ark) induces significant high levels of CD4+, CD8+ and IgA bearing B cells in the trachea of birds [86]. Despite the capacity of live attenuated vaccines to induce both humoral and cellular immunity, they face the challenge of risk of spreading live vaccine virus. Therefore, other types of vaccines such as inactivated vaccines and/or DNA vaccines are considered as alternatives. Inactivated vaccine induces weak cellular immune response and requires priming with DNA vaccines. Priming with DNA vaccines encoding IBV structural genes and boosting with inactivated vaccines induce CD4+CD3+ and CD8+CD3+ T lymphocytes and memory B cells marked with high titers of IgY anti-IBV antibody [87]. More so, IBV-CS vaccine an inactivated IBV vaccine encapsulated in chitosan nanoparticles induced production of IFN-γ, IgA and IgY against IBV [88] and this is unlike the conventional inactivated vaccine, which only elicits limited mucosal immune responses. A combination of live attenuated vaccine and inactivated vaccine containing a BR-I IBV strain confer effective immune protection against infectious IBV strain through the induction of IgY anti-IBV antibodies and effector TCD8 cells and granzyme A [89]. It is believed that the induced anti-IBV IgY was as a result of the development of memory B lymphocytes after administration of live attenuated vaccine a day before re-vaccination with the oil adjuvanted inactivated vaccine, which upregulated IgY gene expression [89]. Full dose vaccination with H120 a live attenuated vaccine on a day-old chick showed full protection against IBV marked with high levels of IBV-specific IgY, IgA and cell-mediated immune genes including, IFN-γ, CD8+ T cells, and granzyme homolog A [82]. This suggests that for complete protection against IBV full dose vaccination is paramount. However, a study reported delayed production of IgA and IgY was observed in day-1 > day-7 > day-14-old chicks when vaccinated with live attenuated Arkansas Delmarva Poultry Industry-type (ArkDPI) IBV vaccine [90]. The authors, therefore, suggested vaccination to be carried out after day-7 post-hatch as the IgY antibodies from the day-1-old chicks had lower affinity and poor vaccine-mediated protection against IBV. More so, vaccination with an attenuated ArkDPI vaccine, elicited low systemic and mucosal antibody responses on day-1-old chicks compared to chickens vaccinated at a later stage in life. According to Saiada et al. [91], the populations of (CD)4+, CD8+, and CD4+/CD8+ T-cells increased with age and this pattern does not change with IBV vaccination. Additionally, ArkDPI vaccines induced greater serum antibodies, B and T-helper cells (CD3+CD4+) and cytotoxic T cells (CD3+CD8+) on day-7 chicks compared with day-1-old chicks [92]. Another study showed that vaccinated chickens that presented high monocyte MHC II expression had the weakest vaccine-induced protection against IBV [93]. In the study, vaccine-induced MHC-II expression correlated with the viral load and response to IBV infection/vaccine varies among the MHC-B haplotypes, with some haplotypes being more resistant compared to the others [93]. Therefore, genetics could play a significant role in infection susceptibility/resistance and monocyte MHC II expression in vaccinated birds could serve as a marker to determine the protective effect of IBV vaccines. The use of DNA vaccines has been shown to induce both humoral and cell-mediated immune responses and could provide for complete protection against IBV infection. A chimeric multi-epitope DNA vaccine induced the production of antibodies, CD4+CD3+, and CD8+CD3+ T-lymphocytes in vaccinated birds [94]. A poly-epitope DNA vaccine consisting of B and T cell epitopes activated naïve B cells to produce neutralizing antibodies and elicited CD8+ T cells (CTL) response against IBV [8].

#### 2.3.2. Vaccine-Mediated Immunity against NDV

NDV live attenuated vaccine (G7M) generated by reverse genetics induced high T-cell proliferation, IFN-γ, and antibodies [95]. VG/GA Newcastle live vaccine induced IgY and IgA anti-NDV antibody responses in vaccinated birds [96]. More so, VG/GA induced strong type I IFN (IFN-α and IFN-β) response in vaccinated chickens prior or post-NDV infection [97]. Inactivated NDV vaccine induced high levels of IL-6 and IFN-γ in vaccinated birds [98]. Vaccination with NDV attenuated vaccine (Nobilis ND LaSota; Cevac Vitapest L) and inactivated vaccine (Nobilis Newcavac) induced the production of anti-NDV IgY, IgM, and IgA in vaccinated birds [99]. NDV DNA vaccine encapsulated in N-2-hydroxypropyl trimethyl ammonium chloride chitosan (N-2-HACC) and N, O-carboxymethyl chitosan (CMC) nanoparticles induced IL-2, IL-4, IFN-γ, anti-NDV IgY and IgA antibodies in immunized birds [100]. NDV DNA vaccine encapsulated in Ag@SiO2 hollow nanoparticles (pFDNA-Ag@SiO2-NPs) induced IL-2 and IFN-γ in vaccinated birds [101]. More so, high titers of serum antibody were induced by NDV/LaSota-N-2-HFCC/CMC-NPs vaccine, and this vaccine significantly promoted the proliferation of lymphocyte and induced high levels of IL-4, IL-2, and IFN-γ in immunized birds [102]. The use of virus-like particle (VLPs) in vaccine production has proven useful as it could elicit both humoral and cell-mediated immune responses in immunized host. Moreover, VLPs mimics the structure of the wild-type virus and could be recognized by the host immune system. For instance, ND-VLPs stimulated the maturation of dendritic cells, upregulated the expression of MHCII, CD40, CD80, and CD86 and cytokine secretions including- TNF-α, IFN-γ, IL-6, IL-4 and IL12p70 in mice. The induction of IgY response and the presence of CD4+, CD8+ T cells indicate the efficiency of VLPs in inducing humoral and cellular immune response [62].

Considering NDV-IBV co-infection in birds, several studies considered developing NDV-IB chimeric vaccines and these vaccines have been demonstrated to be useful in combating mixed infections as shown by their ability to elicit both humoral and cellular immune responses in vaccinated birds. An N-2-HACC-CMC/NDV/IBV NPs and N-2-HACC-CMC/NDV-IBV NPs antigens (NDV and IBV) encapsulated with chitosan induced higher titers of IgY and IgA anti-IBV and anti-NDV antibodies in chickens, promoted significantly the proliferation of lymphocytes, induced high production of cytokines; interleukine-2 (IL-2), IL-4 and interferon-γ (IFN-γ) production in vaccinated chickens. Increased levels of IFN-γ and IL-2 in the study was said to be as a result of higher induction of Th1 responses [103]. Chimeric infectious IB-ND-VLPs vaccine-induced anti-IBV and anti-NDV antibodies, T-cell cytokines, including- IL-4 and IFN-γ and this shows that chimeric IB-ND VLPs can evoke both Th1- and Th2-type cellular immune responses against IBV and NDV infections [104].

### 2.4. Immunopathology in the Hosts Resulting from IBV and NDV

#### 2.4.1. Immunopathology in the Hosts from IBV

IBV causes severe lesions in the kidney [105], air sac [106] and trachea [107] in infected birds. Upon infection, IBV activates the Endoplasmic reticulum (ER) stress response and induces pro-inflammatory cytokines and apoptosis through its M protein. IBV M protein upon glycosylation enhances the activation of GRP78, an ER stress marker, which in turn activates PERK/IRE/CHOP/XBP1 for subsequent trigger of pro-inflammatory cytokines (IL-6 and IL-8) and apoptosis [108]. IBV distorts eggshell formation by reducing the expression of collagen type I gene in the thymus and CaBP-D28K in the uterus (genes related to eggshell formation in those regions, respectively) [109]. According to the authors, a marked infiltration of cytotoxic cells ((CD8+ and TCR-γδ+ T cells), cytotoxic substances (B-NK, perforin and granzyme) and pro-inflammatory cytokines was observed in the mucosa of the IBV-infected chickens [109]. Pro-inflammatory cytokines—IL-1β, IL-6 and IFN-γ—are said to play a major role in tracheal lesions in IBV-infected birds and the induction of CD8αα and Granzyme homolog A gene provides for protective immune response [110]. IBV-Beaudette induced apoptosis in chicken macrophage HD11 cells by activating caspase-8 and caspase-9 pathway through Fas/FasL and increased expression of Bax/reduced expression of Bcl-2, respectively [111]. More so, apoptosis is induced by IBV through the upregulation of pro-apoptotic growth arrest and DNA damage-inducible protein (GADD153) for its downstream function via the ER stress response pro-apoptotic pathways; protein kinase R-like ER kinase (PERK), eIF2α, activating transcriptional factor 4 (ATF4) pathway and protein kinase R (PKR) [112]. IBV infection also triggers the expression of p38 mitogen-activated protein kinase (MAPK) pathway, which also induces production of pro-inflammatory cytokines (IL-6 and IL-8) [113]. MAPK is also responsible for regulation of apoptosis in IBV and IBV infection induce phosphorylation of MAPK kinases 7 (MKK7) which induce the activation of JNK. Activated JNK promotes apoptosis in IBV-infected cells through modulation of Bcl2 family proteins or as a result of cell cycle arrest at the S and G2/M phases in IBV-infected cells [113,114]. It is important to note that although apoptosis is a non-specific defense mechanism that interfere with the replication of viruses in infected cells, it may cause tissue damage as a result of the premature destruction of the infected cells [51,111] and induce acute inflammatory responses and the inflammatory reactions may expose the host to bacterial infections [110]. The level of pro-inflammatory response depends on the IBV strain and the genetics of the infected host [93]. With the influx of pro-inflammatory proteins, the action of CD8+ T-cells and NOs produced by macrophages and/or dendritic cells in response to viral infections could induce severe lesions in infected hosts.

#### 2.4.2. Immunopathology in the Hosts from NDV

Inflammatory responses induced by NDV in infected hosts usually leads to cellular apoptosis and tissue damage. NDV upon infection induce the secretion of high mobility group box 1 (HMGB1) that promotes the production of inflammatory cytokine storm. HMGB1 binds TLR2/4 and RAGE leading to downstream NF-κB activation and cytokine production. More so, the HMGB1-RAGE interaction induced by NDV promotes the activation of ERK1/2 and JNK [115]. Oviductal dysfunction and reduced egg production observed in birds challenged with velogenic NDV genotype VIId was attributed to the excessive release of inflammatory cytokines, chemokines, lymphocyte infiltration, apoptosis, and severe pathological lesions in the oviduct of egg-laying hens [116]. The severity of infection of NDV in bursa of Fabricius was likened to the level of induction of pro-inflammatory cytokines, chemokines, apoptosis, macrophage infiltrations and oxidative stress (as a result of lipid peroxidation caused by nitric oxide released by macrophages). Oxidative injury and tissue damage caused by reactive oxygen and nitrogen species in NDV-infected cells is as a result of influx of phagocytic cells and release of pro-inflammatory cytokines in infection sites made worse by nutritional deficiency [117]. The severity of the pathological condition caused by NDV in infected hosts also depends on the NDV strain and is often marked with depletion in IgM+, infiltration of macrophages, the release of NOs (oxidative stress), infiltration of pro-inflammatory cytokines, chemokines and subsequently apoptosis [118]. Excessive production of IL-1β magnifies the inflammatory storm in NDV-infected hosts. NDV activates the oligomerization-domain leucine-rich repeats containing the pyrin domain 3 (NLRP3) inflammasome and caspase-1 cleavage in infected cells inducing the production and maturation of IL-1β, which magnifies the inflammatory damage in the host [61,119]. One of the studies reported that viral RNA alone is capable of inducing high amounts of IL-1β [61]. According to Li et al. [120], sphingosine-1-phosphate-1 receptor (S1PR1) overexpression also causes increased virus-induced IL-1β and excessive production of pro-inflammatory cytokines. Therefore, an appropriate amount of IL-1β is required to reduce viral replication as excessive amounts may induce inflammatory responses and/or lesions. Moreover, NDV trigger apoptosis in infected cells by upregulating the unfolding protein response (UPR) signaling (PERK-eIF2α, ATF6, and IRE1α), reduce anti-apoptotic genes and activate pro-apoptotic and inflammatory response proteins [121]. ISG12, an interferon-stimulated gene in chickens also stimulates apoptosis in NDV-infected cells to limit viral replication [122]. Necrosis and breakdown of collagen in the spleen of infected birds was as a result of the disruption of the extracellular matrix molecular composition/integrity and the upregulation of matrix metalloproteinase (MMP)-13 and 14 in NDV-infected cells [123].

## 3. Vaccination against IB and ND

### 3.1. Available Vaccines against IB and ND

#### 3.1.1. Available Vaccines against Infectious Bronchitis

Vaccination has remained the main strategy for controlling infectious bronchitis (IB) in poultry. However, preventive measures against IB are usually hindered by the vast antigenic variations resulting from continuous mutation in the spike protein (S) of the IB virus (IBV) [8]. This continuous mutation leads to different circulating serotypes/genotypes, which usually do not provide cross-protection. The best protective effect is suggested to be achieved by using vaccines targeting only strains of the same genotype [124]. Therefore, this calls for the continuous development of several vaccine types against IB (Table 1) based on the circulating strains or serotypes of IBV. Moreover, considering the cost of vaccine production and the prolonged time required for approval, developing a new vaccine against new IBV variants is usually not always seen as an option. More so, the reported live attenuated vaccines against IB require lengthy time with several passages in embryonated chicken eggs, without any details on the mechanism of attenuation (Table 1). Therefore, live attenuated vaccines are faced with the uncertainty of vaccine safety on long-term usage and the challenge of vaccination with multiple strains of live vaccines is compounded by possibilities of recombination of live vaccine strains with field strains [125]. Furthermore, to provide a broader cross-protection, vaccination with two or more antigenically diverse vaccines is often employed [126]. Generally, live attenuated vaccines may revert to virulence and may induce clinical signs, lesions, ciliostasis, and could lead to the development of secondary infections (Table 1). Inactivated vaccines, on the other hand, is faced with the challenges of low immune evocation and often requires priming with live vaccines and/or adjuvants to boost immune response (Table 1). These call for serious development and improvement on vaccines against IB in poultry as vaccination remains the gold standard for IB prevention.

#### 3.1.2. Available Vaccines against Newcastle Disease (ND)

For effective vaccination against ND, it is important to consider the antigenic similarity between the vaccine strain and the prevalent strain of NDV. Although all NDV strains are of one serotype, their genetic diversity is quite vast and may not provide complete cross-protection [144]. Moreover, for effective vaccination, it is important to reduce shedding of the virulent virus, which may not be provided for by vaccine strains from another genotype. Therefore, due to the antigenic variation, reduced protection is observed from one genotype to another, and inefficiently vaccinated birds could serve as reservoirs and hence, the continual spread of infection [144]. This requires the continuous monitoring and evaluation of the efficacy of vaccines against prevalent and/or newly isolated strain, which is paramount for effective ND prevention and control. Several candidates live attenuated vaccines applied reverse genetics and fusion (F) protein cleavage site to generate genotype matched vaccines (Table 2). However, it is not yet known which F protein cleavage site is best to generate a genetically stable, safe and effective vaccine [145,146], as mutated F protein cleavage site based inactivated vaccine strains might revert to virulent strains. Generally, most of the vaccines reported (Table 2) are live attenuated vaccines and/or inactivated vaccines. Live attenuated vaccines are faced with certain safety concerns; the possibility of reversion to virulence, the need for biocontainment during production and cold-chain requirements [147]. Inactivated vaccines, on the other hand, are faced with poor immunogenicity and if not completely inactivated, may cause disease [147]. Therefore, the need for improvement in ND vaccine development and vaccination cannot be overemphasized.

### 3.2. Recent Advances in Vaccine Development against IB and ND

#### 3.2.1. Recent Advances in IB Vaccine Development

In recent times different studies have developed novel vaccine candidates different from the traditional approaches in a bid to find lasting solutions to IB prevention and control. It is thought that several epitope peptides from B-cells and T cells conserved among several strains can be combined to evoke both humoral and cytotoxic T lymphocytes (CTL) immune response reducing the challenges in the use of a live attenuated vaccine in the control of IB in poultry [160]. In designing an epitope-based vaccine, it is important to predict and screen for the functional neutralizing B-cell and species-restricted T-cell epitopes. This has been proposed to represent a novel strategy to vaccine development against IBV infection in poultry [160]. These suggestions were based on the observed prediction of the B-cell and T-cell epitopes within the S1 glycoprotein of M41 and CR88 IBV [160]. Moreover, Tan et al. [8] constructed a poly-epitope based DNA vaccine pV-S1B+S1T based on recombinant (S1 subunit protein [pV-S1B], a combination of neutralizing epitopes and BF2- restricted T cell epitope box [S1T]). This vaccine elicits strong humoral and cellular immune response against the challenge strain, has no risk of gene integration into the host, and does not trigger an infection in the course of immunization [8]. A novel vaccine candidate based on self-assembly protein nanoparticle (SAPN) reportedly elicited high immune response, reduced tracheal virus shedding with lesser tracheal lesions [161]. IBV-Flagellin-SAPN was developed by the repeated display of the heptad repeat (HR) regions of B-cell epitopes of IBV S2 proteins in their native form. The bioactive domain of flagellin was co-displayed to self-adjuvant the vaccine [161]. A self-adjuvanted vaccine peptide-based vaccine provides for improved immunogenicity with a reduced cost of vaccination. More so, considering that IBV S2 proteins are highly conserved, it is thought that S2-derived peptides with S2 proteins could serve as a marker-based antigen for the development of broad-based vaccines as it reportedly reacts with sera against different IBVs [162]. According to these authors, the 16R amino-acid of IBV S2 protein is suggested as the key amino-acid mediating the antigenicity of S2 protein.

#### 3.2.2. Recent Advances in ND Vaccine Development

DNA vaccines have emerged as a novel strategy for vaccination against ND infection. It is thought to be safe, genetically stable, convenient, and immunogenic [147]. However, following the challenges of DNA vaccines including low bioavailability and degradation before reaching the antigen-presenting cells (APCs), Gao and co, developed a DNA vaccine delivery system made up of copolymer of poly (lactide-co-glycolide acid) and polyethylene glycol (PLGA-PEG-PLGA) hydrogel, encapsulating the recombinant NDV hemagglutinin-neuraminidase (HN) plasmid. They achieved gradual and sustained release of the plasmid from the hydrogel, improving the biological activity. According to the group, the NDV DNA hydrogel vaccine not only provided a 100% protection, but it also enhanced both humoral and cellular immunity against a highly virulent NDV (F48E9 strain) [147]. Similarly, earlier studies by some other groups demonstrated the effectiveness of N-2-hydroxypropyl-trimethyl ammonium chloride chitosan (N-2-HACC) and N, O-carboxymethyl chitosan (CMC) (N-2-HACC-CMC) and chitosan (CS)-coated poly (lactic-co-glycolic) acid (PLGA) nanoparticles (NPs) as an immune delivery carrier for DNA vaccines [100,163,164]. A subunit vaccine based on the NDV F protein was developed by expressing the F protein in Pichia pastoris, this vaccine candidate adjuvanted with flagellin (FliC) stimulated both humoral and cellular immune response against the challenge strain [165]. Moreover, recent findings suggest DNA prime-protein boost vaccination approach applying the full-length NDV F gene to provide an enhanced immune response against NDV in poultry [166]. A transgenic rice ND subunit vaccine reportedly elicited neutralizing antibodies against both homologous and heterologous NDV strains [167]. This plant-produced F vaccine was developed based on Oryza sativa transgenic F protein expressed from transgenic rice seeds, and expression level increased through the hybridization of F-transgenic rice to low-gluten rice [167]. In the development of the plant-produced F vaccine, differentiating infected from vaccinated animals (DIVA) was considered. It is thought to be a strategy and eradication plan for ND control because of the dissimilarity in the antibody profiles of the vaccine and the prevalent strains [167]. Unfortunately, with live attenuated vaccines and inactivated vaccines, DIVA cannot be met. This is because attenuated and inactivated vaccines, having the full components of the virus antigens produce antibodies that are similar and indistinguishable from those produced by infecting viruses. However, DIVA should be considered in the production of other subunit vaccines because of the obvious advantage it provides in segregating infected from vaccinated birds. Moreover, a group earlier expressed the F and HN epitopes of NDV in tobacco seedlings and demonstrated that the transgenic plant extract induced immune responses [168]. This suggests plants as useful vectors for epitope-based vaccines and could provide for the advantages of easy oral route vaccination and induction of mucosal immune response.

### 3.3. IB and ND Vaccine Development

To solve the challenges of IB and ND co-infection in poultry, several studies developed a bivalent vaccine against IBV and NDV challenge. rLaSota-S1 a bivalent vaccine candidate developed with a recombinant LaSota strain and the S1 gene of LX4 IBV induced NDV hemagglutinin inhibition (HI) antibodies, IBV-specific IgG antibodies and cellular immunity against challenge with virulent NDV and IBV in vaccinated chickens [169]. Some other studies reported a recombinant NDV expressing IBV S protein generated using reverse genetics technology to fully protect from challenge with virulent NDV and IBV [170,171]. Similarly, an rNDV-IBV-T/B multiple-epitope NDV vectored vaccine developed using reverse genetics, protected against IBV and NDV [172]. More so, R-H120-HN/5a an IBV/NDV recombinant vaccine developed by reverse genetics reportedly induced humoral immune response and provided protection against challenge with virulent IBV and NDV. In the construct, the 5a gene of recombinant H120 was replaced with LaSota HN gene [173]. Herpes of turkey (HVT) is an alphaherpesvirus that belongs to the *Mardivirus* genus as Marek virus. In contrast to the Marek virus, HVT is nonpathogenic in chickens and have been used successfully for protection against marek disease for years [174]. Recombinant HVT (rHVT) are produced by inserting a foreign gene that encodes a specific protein from another virus in the HVT vector [175]. rHVT vaccines have been used for protecting against Marek virus and many other avian diseases and have been used successfully against IBD [176,177] and ND [178,179]. Owing to the challenges faced with live attenuated vaccines and inactivated vaccines as earlier discussed, the use of virus-like particles (VLPs) is thought to be a promising approach to vaccine development against IB and ND infection. VLPs are non-infectious, empty sheets of virus structural proteins with similar morphology as the native virus [104]. An IB-ND virus-like particles (IB-ND VLPs) vaccine reportedly induced both humoral and cellular immune responses specific for IBV and NDV without requiring adjuvants [104]. In the same study, the IB-ND VLPs vaccine was developed by linking the recombinant F (rF) protein of NDV and the recombinant S (rS) protein of IBV and IBV M protein through the Baculovirus system.

## 4. Conclusions

Chicken and other poultry have robust immune systems against viral diseases. This is usually exploited for vaccination against IBV and NDV. Since these two viruses are most deadly in young birds, vaccination has to be given early. It has been shown that both dosage and timing are critical for the success of vaccination. Some level of success has been achieved with both IB and ND vaccination. However, both viruses undergo rapid mutations that lead to the emergence of new circulating viral strains, which require the continuous development of new vaccines. This makes the control of these two viruses very challenging and expensive. The emergence and advancement in recombinant DNA technology have opened novel avenues for potent vaccine development with possibilities for having bivalent, multivalent, and/or self-adjuvanted vaccines. Subunit vaccines against IB and ND developed through this technology require only the immunogenic portion of the target virus displayed on the surface of a backbone structure, solving the risk of recombination and reversion to virulence often encountered with live and inactivated vaccines. It addresses most of the safety issues as they lack the viral RNA, while still stimulating immune response like the native virus as its structure and morphology match that of the infectious virus as seen in the VLPs discussed [104]. Development of new vaccines using this technology will make DIVA possible, thus making surveillance and monitoring easier. Additionally, the development of bivalent vaccines against IBV and NDV offers the opportunity to tackle these two menaces at the same time and, as the popular saying goes, brings the possibility of “killing two birds with one stone”. This means using one vaccine to tackle two dangerous viruses, thereby setting the birds free. Finally, this review has established that a more comprehensive and successful control of both IBV and NDV through vaccination is possible, and this will go a long way to contributing to global food security.

## Figures and Tables

**Table 1:** Available vaccines against infectious bronchitis in poultry.

S/N	Vaccine Name	Type of Vaccine	Formulation	Parent Strain	Challenge Strain	Observed Response
1	SZ130 [127]	Live attenuated vaccine	Continuous passage in chicken embryo for 130 generations	Attenuated QX-like IBV strain SZ130	QX-like IBV strain SD	Reduces the proliferation efficiency of the challenge strain
2	IBV-CS [88]	Inactivated vaccine	Ionic gelation method (IBV, BR-1 genotype strain encapsulated in chitosan nanoparticles)	IBV, BR-1 genotype strain	IBVPR-05 strain	IgA- and IgY-mediated mucosal immune responses and T-cell-mediated immunity (CMI) responses
3	SczyC100 [128]	Live attenuated vaccine	Continuous passage in chicken embryo kidney cells for 100 times	Attenuated GI-19/QX-like field isolate Sczy3	GI-19/QX and GI-7/TWI type virulent strains	Reduced morbidity, mortality, and tracheal viral loads caused by the challenge strains
4	ArkDPI [129]	Live attenuated vaccine	Passaged 50 times in embryonated eggs and further passaged by individual vaccine companies to generate different commercial ArkDPI-derived Ark serotype IBV vaccines	Attenuated Ark-type IBV	Ark IBV serotype	Induced protection against homologous challenge
5	ArkGA [130]	Live attenuated vaccine	Continuously passaged 60 times in 9-to-11 day-old embryonated chicken eggs	Ark99 vaccine no longer commercially used	Ark IBV serotype	Induces efficacious immune response against the challenge strain
6	ZYYR-2014 [131]	Live attenuated vaccine	Limiting dilution passage attenuation in embryonated chicken eggs (five passages)	Attenuated QX-like IBV field strain ZYY-2014	QX-like (HSJ-2016) and Mass (M41) types.	Provided full protection against the IBV challenge strain
7	K40/09 [124]	Live attenuated vaccine	Continuous passage in embryonated eggs for 50 times at sub-optimal higher temperature (56 °C)	Attenuated K40/09 strain	KM91-like and QX-like subgroup	Elicited high titers of neutralizing antibodies against the challenge strains.
8	YX10p90 [132]	Live attenuated vaccine	Continuous passage in fertilized chicken eggs for 90 times	Attenuated IB, QX-like YX10 strain	YXp5, CHI, CHIII, and CHV genotypes	Showed 100% protection against YX10p5, effective protection against CHI genotype strains, and partial protection against CHIII and CHV genotype strain
9	K2p170 [133]	Live attenuated vaccine	Continuous passage in embryonated chicken eggs for 170 times	Attenuated K2/01 strain	KM91-like and QX-like subgroup	Produced neutralizing antibodies against challenge strains and provided almost complete protection
10	Variant 2 [134]	Inactivated vaccine	Inactivated after 10th passage in embryo with formaldehyde, passaged 3 times after inactivation and emulsified	Variant 2 (IS-1494/GI-23) genotype	IBV variant 2 viruses	Elicited neutralizing antibodies against the challenge strain
11	att-IBM41 [135]	Live attenuated vaccine	Continuous passage in embryonated chicken eggs for 100 times	Attenuated IBM41 strain	IBM41 strain	Reduced viral shedding
12	att-IB2 [135]	Live attenuated vaccine	Continuous passage in embryonated chicken eggs for 100 times	Attenuated IB2 strain	IBM41 strain	Reduced viral shedding
13	ME VAC IB-VAR2 [135,136]	Live attenuated vaccine	Continuous passage in embryonated chicken eggs for 110 times	Attenuated variant 2 IBV strain Eg/1212B/2012	Variant 2 strains (Egy/VarII	Reduced viral shedding, Produced neutralizing antibodies against the challenge strain
14	GA08/GA08HSp16/08 [137]	Live attenuated vaccine	Continuous heat treatment (56 °C) passage in embryonated chicken eggs for 8 times, followed by final passage for 4 times without heat treatment	Attenuated GA08/pass4/08 strain	GA08/pass4/08 strain	Induced protective immune response against challenge strain
15	IB H120 [138]	Live attenuated vaccine	Passage in embryonated chicken eggs for 120 times	Attenuated mass type IB H strain	Recommended for IBV Massachusetts type	Stimulates both humoral and cellular immune responses to challenge strains
16	LDT3-A [139]	Live attenuated vaccine	Continuous passaging in chicken embryos for 120 times	Attenuated tl/CH/LDT3/03 strain	tl/CH/LDT3/03 strain	Provided protection against challenge strain
17	GI-19 (SZ200) [140]	Live attenuated vaccine	Continuous passage in embryonated chicken eggs for 200 times.	Attenuated GI-19 vaccine strain SZ200	GI-19 and GI-22 genotype strains	Provided protection against the challenge strain
18	rH120-S1/YZ [141]	Live attenuated vaccine	Reverse genetics, S1 gene of H120 vaccine strain replaced with that of ck/CH/IBYZ/2011	H120 vaccine strain	rIBYZ a QX-like strain	stimulated both humoral and mucosal immunity to the vaccinated birds
19	BeauR-4/91(S) [142]	Live attenuated vaccine	Reverse genetics, the ectodomain region of Beaudette S glycoprotein replaced by that of IBV 4/91	Beau-R strain	IBV 4/91 and M41-CK strain	Provided protection against challenge with 4/91 but less protection against IBV M41
20	IB TW [143]	Live attenuated vaccine	Passage in specific pathogen-free (SPF) embryonated eggs 74 times	IBV strain 2575/98 a Taiwan Group I strain (TW I)	IBV strain 2993/02 (TW I)	Provided 90% protection against challenge with pathogenic field IBV 2993/02 and stimulated neutralization antibodies with neutralization index greater than 4.4

**Table 2:** Available vaccines against Newcastle disease in poultry.

S/N	Vaccine Name	Type of Vaccine	Formulation	Parent Strain	Challenge Strain	Observed Response
1	NDRL0901 [148]	Live attenuated vaccine	Passaged six (6) times in specific pathogen-free (SPF) chickens	DK1307 strain of duck origin	Kr005 strain	Showed >80% protection from mortality irrespective of administration route.
2	ND I_2_ [149]	Thermostable Live vaccine	Passaged two (2) times in the allantoid cavity of embryonated chicken egg.	NDV I_2_ strain	Local NDV strain	Induces antibody response and protective immunity against challenge strain
3	VG/GA [150]	Live attenuated vaccine	Passaged three times in embryonated chicken egg.	VG/GA strain	NDV GB strain	Provided 95–100% protection against challenge strain
4	LaSota and PT3 [151]	Live vaccine	Lentogenic live strains	LaSota strain and PT3 strain	NDV SD strain, NDV DY strain	Provided full protection against challenge strains
5	NDV/A14 [152]	Inactivated vaccine	Amino-acid sequence of the cleavage site of the F0 protein was changed by reverse genetics, passaged 10 times in chicken embryo and inactivated with 0.7% formaldehyde	NDV genotype VII JS3/05 strain	NDV genotype VII JS3/05 strain	Reduced viral shedding and protected the birds against clinical disease
6	Ban/AF [153]	Live attenuated vaccine	Virulent F protein motif was modified to avirulent motif by reverse genetics and passed 10 times in chicken	NDV strain Ban/010	NDV strain Ban/010	Provided full protection against the challenge virus and prevented viral shedding
7	NDV O/A14 [154]	Inactivated vaccine	HN gene of NDV/A14 vaccine strain replaced with that of JS-14-12-Ch bearing E347K and G362A co-mutation	JS-14-12-Ch HN gene in NDV/A14	JS-14-12-Ch NDV strain	Provided full protection, reduced viral shedding and viral loads.
8	aSG10 [155,156]	Live attenuated vaccine	Reverse genetics and the protease cleavage site of the virulent F0 protein was altered to avirulent strain	SG10 strain	SG10 strain	Provided full protection and reduced viral shedding
9	G7M [95]	Live attenuated vaccine	Reverse genetics and the replacement of F cleavage site sequence typical of velogenic strains with that of LaSota vaccine	G7 strain	Genotype VII NDV (G7 strain)	Provided full protection against the challenged virus strain, induced both humoral and cell-mediated immunity, reduced virus replication and shedding
10	rLS1-XII-2 [157]	Live attenuated vaccine	Reverse genetics, the ecto- and trans membrane domains of the F and HN protein in the rLS1 backbone were replaced with those of PP2011	Genotype XII (PP2011) strain	Genotype XII (PP2011) strain	Provided full protection against the challenged strain and reduced virus shedding
11	rGM-VIIm [146]	Live attenuated vaccine	Reverse genetics, rGM attenuated by changing the F0 polybasic cleavage site to monobasic	Genotype VII GM strain	GM NDV strain	Both inactivated and live rGM-VIIm provided full protection against the challenge strain. However, the live vaccines reduced viral shedding more than the inactivated vaccine.
12	mIBS025 [158]	Live attenuated vaccine	Reverse genetics, in silico modification of F cleavage site from virulent polybasic to avirulent monobasic motif, modified sequence chemically synthesized and inserted into pOLTV5 transcription vector	Complete genome sequence of NDV IBS025/13 strain	NDV strain IBS002/11	Induced strong antibody-mediated immunity and reduced viral shedding
13	rNDV-R2B-FPCS [159]	Live attenuated vaccine	Reverse genetics, changing the F protein cleavage site from polybasic amino acid to dibasic amino acid	rNDV-R2B	Virulent NDV strain (Accession No. KJ769262.1)	Stimulated both humoral and cellular immunity, reduced viral shedding

## Data Availability

No new data were created or analyzed in this study. Data sharing is not applicable to this article.
